# Blinking Fluorescent Probes for Tubulin Nanoscopy in Living and Fixed Cells

**DOI:** 10.1021/acschembio.1c00538

**Published:** 2021-11-04

**Authors:** Ru̅ta Gerasimaitė, Jonas Bucevičius, Kamila A. Kiszka, Sebastian Schnorrenberg, Georgij Kostiuk, Tanja Koenen, Gražvydas Lukinavičius

**Affiliations:** † Chromatin Labeling and Imaging group, Department of NanoBiophotonics, Max Planck Institute for Biophysical Chemistry, Am Fassberg 11, 37077 Göttingen, Germany; ‡ Department of NanoBiophotonics, Max Planck Institute for Biophysical Chemistry, Am Fassberg 11, 37077 Göttingen, Germany; § EMBL Imaging Centre, EMBL-Heidelberg, Meyerhofstr.1, 69117 Heidelberg, Germany

## Abstract

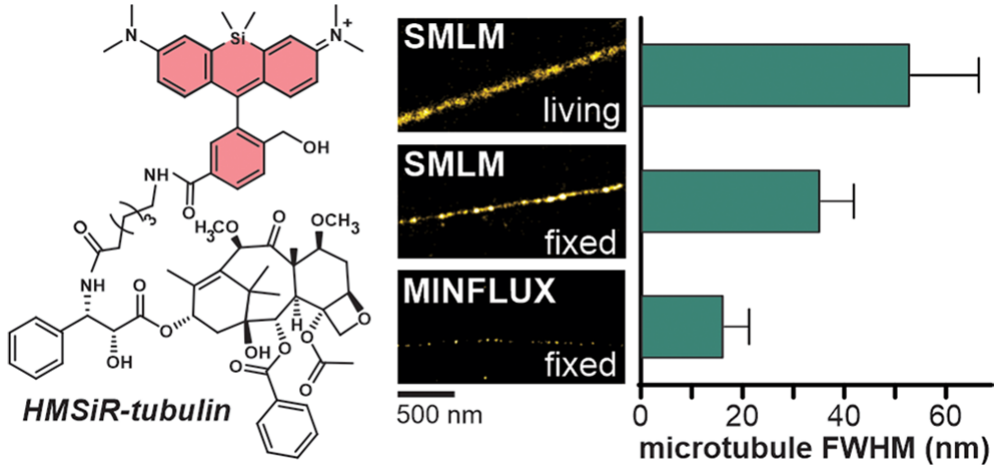

Here we report a small molecule tubulin probe for single-molecule localization microscopy (SMLM), stimulated emission depletion (STED) microscopy and MINFLUX nanoscopy, which can be used in living and fixed cells. We explored a series of taxane derivatives containing spontaneously blinking far-red dye hydroxymethyl silicon–rhodamine (HMSiR) and found that the linker length profoundly affects the probe permeability and off-targeting in living cells. The best performing probe, HMSiR-tubulin, is composed of cabazitaxel and the 6′-regioisomer of HMSiR bridged by a C6 linker. Microtubule diameter of ≤50 nm was routinely measured in SMLM experiments on living and fixed cells. HMSiR-tubulin allows a complementary use of different nanoscopy techniques for investigating microtubule functions and developing imaging methods. For the first time, we resolved the inner microtubule diameter of 16 ± 5 nm by optical nanoscopy and thereby demonstrated the utility of a self-blinking dye for MINFLUX imaging.

## Introduction

As one of the major components of the cytoskeleton, tubulin is involved in trafficking of biomolecules, cell movement and division. Paclitaxel is one of the most effective anticancer drugs used for the treatment of solid tumors such as ovarian, breast, and lung cancers.^1^ It acts by stabilizing microtubules and thereby blocking cell progression through mitosis. It was originally isolated from the bark of the yew tree *Taxus brevifolia* in 1971,^2^ and a tremendous repertoire of analogues has been reported by research groups and pharmaceutical companies ever since.^1,3^ Some of these analogues found their use in microscopy applications. Flutax-1 and Flutax-2 were the first fluorescent probes for tubulin imaging.^4,5^ They are composed of paclitaxel and fluorescein or Oregon Green, respectively. Further developments encompass various taxane analogs coupled to red fluorescent dyes, such as boron-dipyrromethene (BODIPY) dyes, rhodamines, coumarins, or carbo-, germano- or silicon-rhodamines.^4,6−10^ All these probes are designed for ensemble fluorescence microscopy methods. The development of tubulin probes for single-molecule localization microscopy (SMLM) in living cells is however lagging behind, with only one published example composed of a spiropyran derivative and colchicine, which requires UV illumination.^11^ We are bridging this gap by synthesizing taxane analogs coupled to the spontaneously blinking fluorophore hydroxymethyl silicon–rhodamine (HMSiR).^12^

## Results and Discussion

**Figure 1:**
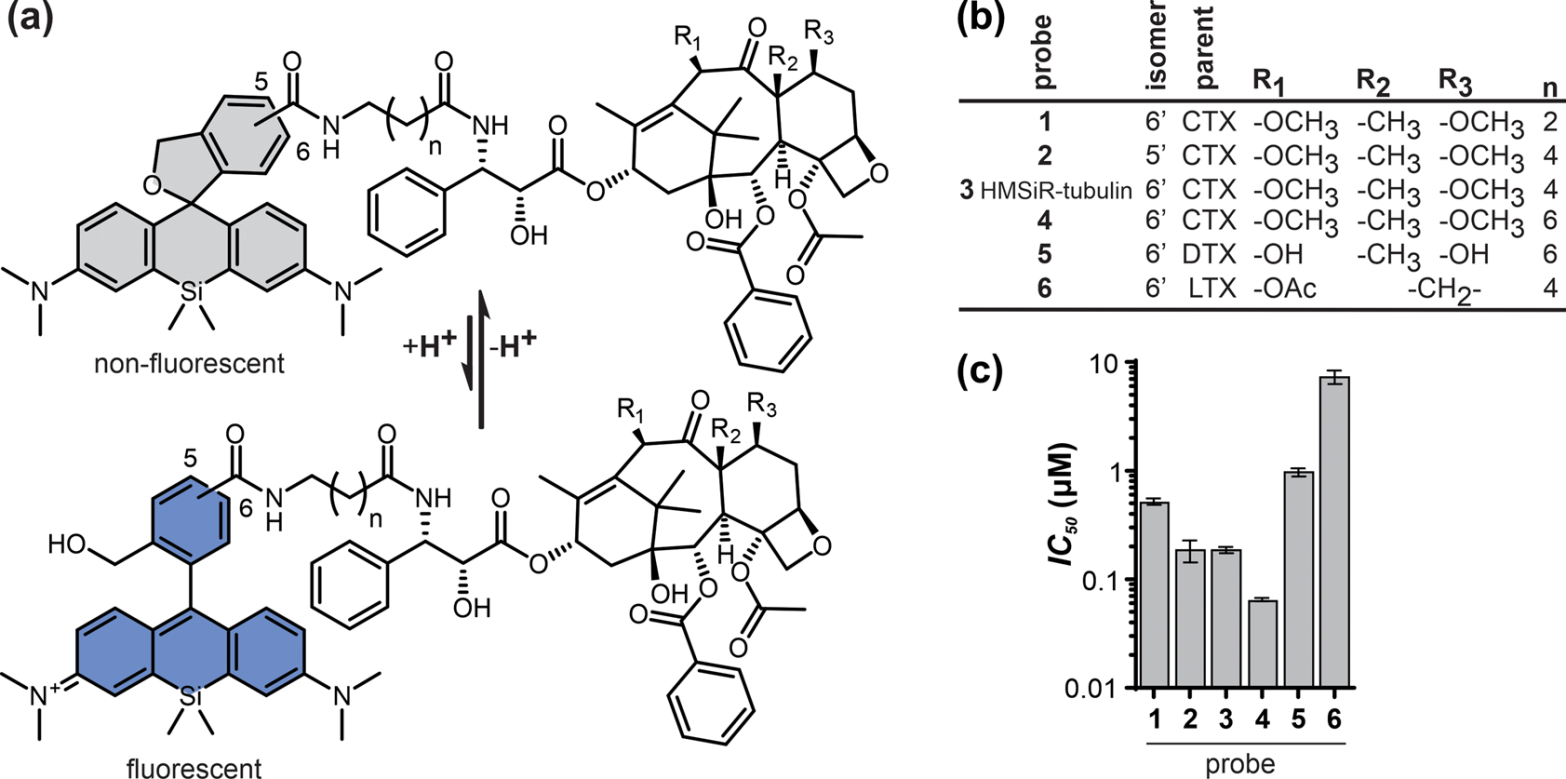
Tubulin probes synthesized in this study. (a) General structure of the probes, showing spirocyclization of the fluorophore that is responsible for spontaneous blinking. (b) Structure and naming convention of the probes. (c) Toxicity in HeLa cells after 24 h incubation with the probes (mean ± SD, *N* = 3).

Our previous work has repeatedly pointed out the importance of finding the optimal combination of a targeting moiety and a fluorophore.^13−15^ Therefore, we explored several designs by coupling the 6′-regioisomer of HMSiR to the often used analog docetaxel (DTX) or taxanes that are poorly recognized by multidrug resistance proteins, cabazitaxel (CTX) and larotaxel (LTX)^16,17^ (Figure 1a,b).

In our preliminary experiments, the CTX-containing probe performed best, and we fine-tuned its structure by varying the linker length and introducing the 5′-regioisomer of HMSiR.

The 5-HMSiR-COOH and 6-HMSiR-COOH regioisomers were obtained by previously described procedures.^12,18^ The design of tubulin probes is based on the replacement of the *tert*-butyloxycarbonyl (Boc) group in taxanes with HMSiR tethered via linkers of variable length at the 3′-position (Figure 1a,b). This was achieved by performing peptide coupling reactions with 5/6-HMSiR-COOH dyes and taxanes bearing the linker with a terminal amino group (Scheme S1) or by attaching required linkers with a terminal carboxylic acid group to the HMSiR moiety and further coupling it to *N*-*desboc*-taxane derivatives (Scheme S2).

To get insight into the probes’ performance *in cellulo*, we stained U-2 OS cells and imaged them with a spinning disk confocal microscope. In living cells, microtubule staining by probes **5** and **6** was weak, largely overshadowed by bright staining of cytoplasmic vesicles (Figure S1a). In contrast, all CTX-based probes stained mainly microtubules, and probe **3** (HMSiR-tubulin) was the brightest. In none of the cases did addition of verapamil^13,14^ have an effect, indicating that efflux pumps do not limit probe accumulation or staining efficiency. Fixing cells with 0.2% glutaraldehyde followed by quenching with NaBH_4_ is known to preserve taxane interaction with tubulin.^19,20^ All probes stained microtubules in fixed cells (Figure S1b), although the intensity varied. Probe **1** stained fixed microtubules as brightly as HMSiR-tubulin (**3**). This indicates that plasma membrane permeability of probe **1** in living cells is suboptimal. The remaining probes were dimmer, suggesting either reduced affinity or unfavorable interaction between fluorophore and tubulin or both. In fixed cells, probes **5** and **6** stained microtubules, indicating that the cell permeability is the major factor compromising their performance. In concordance, probes **5** and **6** showed low cytotoxicity, with half maximal inhibitory concentration (*IC_50_*) in the micromolar range (Figures 1c and S2). In the CTX miniseries, probe **1** was the least toxic, which is fully consistent with the staining data.

We assessed probe toxicity by measuring accumulation of HeLa cells at sub-G1 cell cycle stage after 24 h (Figure 1c, S2). For the best probes, the concentration required for staining was lower than the *IC_50_*; thus no major toxicity is expected during short incubations. However, in each case determining the shortest incubation time and the lowest probe concentration is recommended to minimize probe interference with the interphase processes.^21^

At this point, we concluded that CTX-containing probes hold more promise for *in cellulo* tubulin imaging and focused further investigation on them.

**Figure 2:**
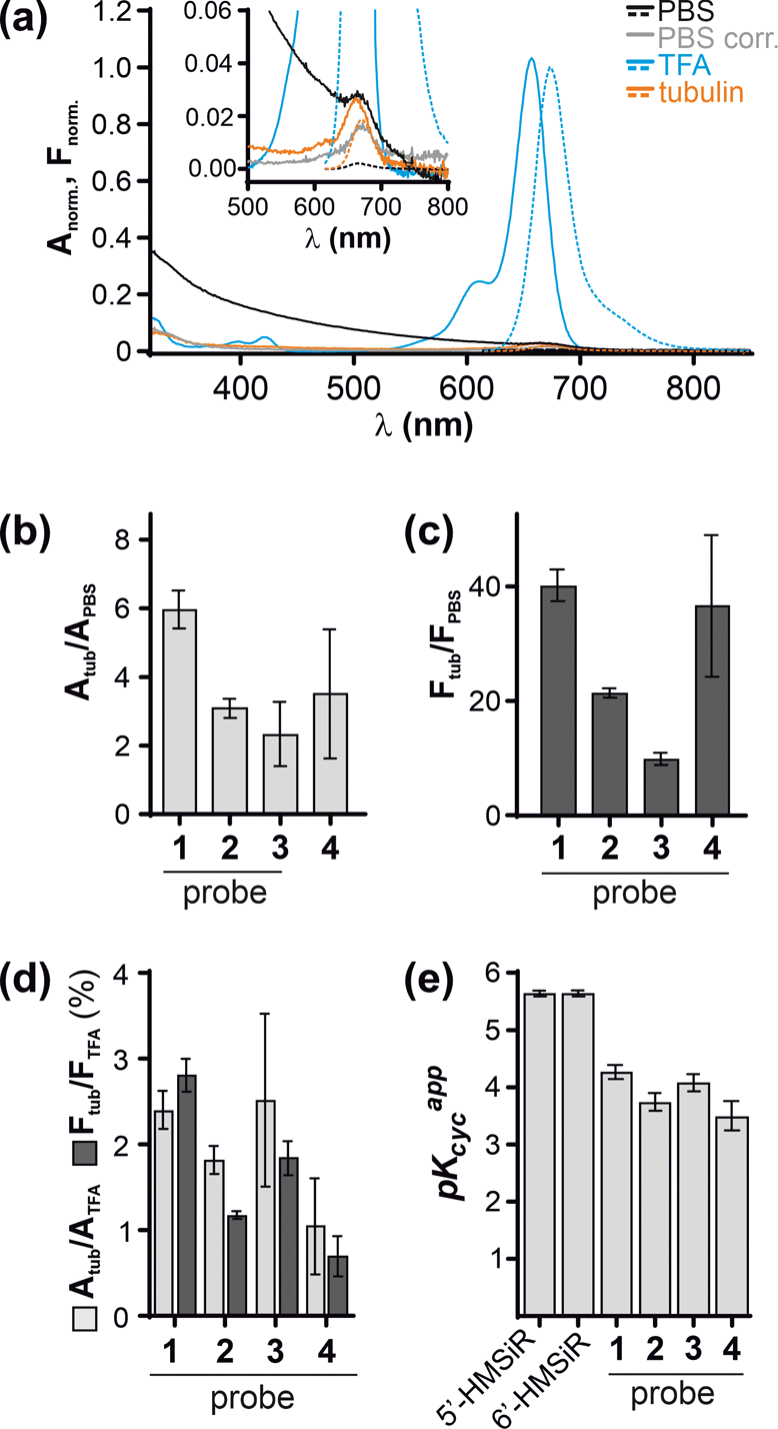
Properties of HMSiR CTX-based probes. (a) Normalized absorbance (solid line) and fluorescence (dashed line) spectra of **3**. PBS corr., absorbance spectrum corrected for light scattering. The inset zooms in on weak signals in aqueous buffers. (b, c) Absorbance (659 nm) and fluorescence (673 nm) increase upon tubulin binding, as compared to PBS. (d) Percent of absorbing and fluorescent probe when bound to tubulin. Maximum values were determined in ethanol + 0.1% TFA; mean ± SD, *N* = 3 or 4. (e) Apparent cyclization constants (pH at which half of molecules are in the spiroether state) of 5′- and 6′-regioisomers of free dyes and HMSiR-CTX probes.

HMSiR dye exists in the equilibrium between fluorescent and nonfluorescent/nonabsorbing states (Figure 1a). This equilibrium is strongly shifted toward the dark spiroether state at physiological pH (∼7.4). Only a small subset of molecules exist in a fluorescent state at a given time point leading to spontaneous appearance of spatially separated light emitters: blinking events.^12^ This equilibrium is highly sensitive to the fluorophore environment, which can change upon target binding. In aqueous buffer (PBS), our probes showed nearly no fluorescence and displayed broad absorbance spectra dominated by light scattering, indicating aggregation (Figure 2a). Binding to tubulin resulted in appearance of fluorescence and absorbance peaks with no signs of light scattering, indicating disassembly of the aggregates. Equilibrium shift upon tubulin binding results in 2–6-fold increase of absorbance and 10–40-fold increase of fluorescence (Figures 2b,c). The changes of absorbance are mainly determined by spirocyclization equilibrium, while fluorescence is additionally quenched by intramolecular interactions in the aggregates. We estimated that 1–3% of the probe exists in the fluorescent form when bound to tubulin by comparing absorbance and fluorescence in the presence of tubulin to absorbance and fluorescence in ethanol + 0.1% TFA, which represents the maximum values (Figure 2d, S3).

In addition to staining microtubules, HMSiR CTX-based probes can accumulate in intracellular vesicles, most likely acidic compartments. This was observed in several cell lines (Figures S1 and S4) and in mouse primary hippocampal neurons (Figure S5). The trend was always the same: probe **4** showed the most and HMSiR-tubulin (**3**) showed the least off-target staining. As weak bases, the rhodamine probes can be protonated and trapped in the acidic vesicle lumen. This can be rescued by increasing luminal pH, for example, by applying millimolar concentration of NH_4_Cl.^22^ Indeed, adding 20 mM NH_4_Cl to prestained human fibroblasts substantially reduced the off-target staining of vesicles (Figure S4). NH_4_Cl treatment did not eliminate the microtubule staining, suggesting that dye switching-off due to alkalinization cannot fully account for the loss of vesicle fluorescence. Importantly, we do not recommend using NH_4_Cl for increasing staining specificity because of the possible disruptive effects on the cell physiology.^23^

Alternatively, low pH can shift the equilibrium toward the fluorescent state thus making the mistargeted probe **4** more visible. We measured probe absorbance at different pH and found that apparent cyclization constant (*pK_cyc_^app^*) of the probes is notably lower than that of the free dye^12^ and, likely, shifted by aggregation (Figures 2e and S6). However, as *pK_cyc_^app^* values were very close for all the probes, with that of probe **4** being the lowest (the most acidic), this mechanism cannot explain more prominent lysosome staining by this probe. Altogether, this data suggests that a long aliphatic linker favors probe mistargeting into acidic vesicles. Interestingly, despite poor staining of microtubules, probe **4** is substantially more toxic than others (Figure 1c), which might result from lysosome damage.

HMSiR-tubulin (**3**) and probe **2** showed the best biocompatibility and staining. Although different only in fluorophore positional isomer, probe **2** consistently appeared less bright in living and (to a lesser degree) in fixed cells. We evaluated the binding affinity of these two probes by measuring the fluorescence intensity of the fixed microtubules at variable probe concentrations. The binding curves could be fitted to a single-site dose response equation (Figure S7). The *K_D_^app^* for both probes were very similar and close to the values (10–100 nM) reported for Flutax probes.^5,20^ We found *K_D_^app^* = 121 ± 8 nM and 115 ± 8 nM, for HMSiR-tubulin (**3**) and probe **2**, respectively. Thus, differences in performance of these two probes do not result from different affinity to tubulin.

**Figure 3:**
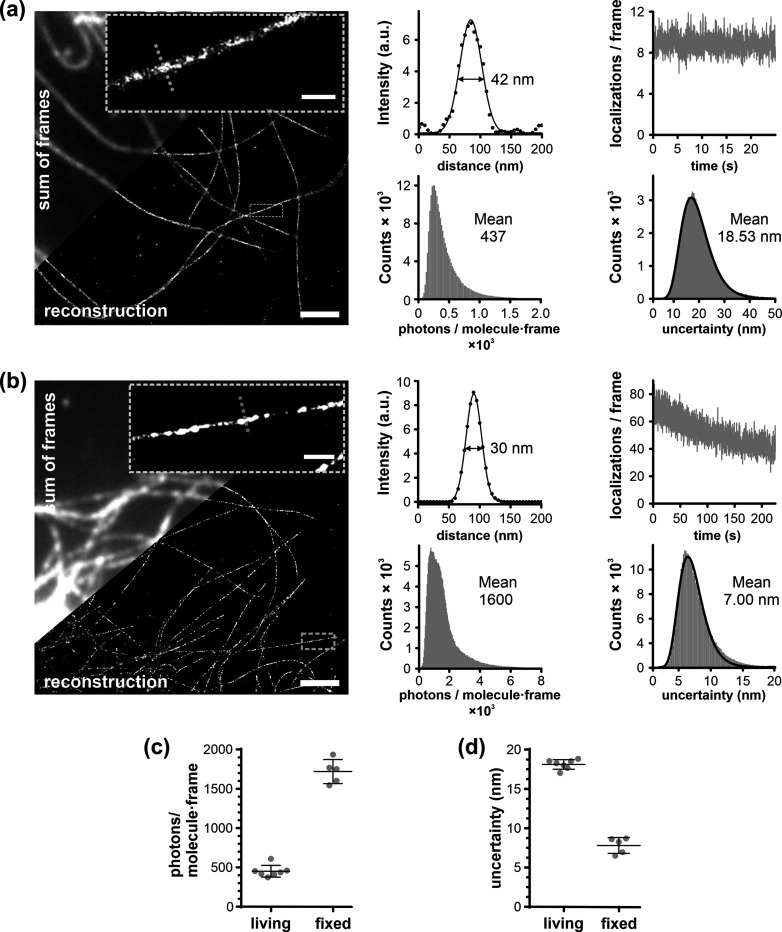
SMLM imaging of microtubules in living and fixed U-2 OS cells stained with HMSiR-tubulin (**3**): (a) Living cells. (b) Fixed cells. Zoom-in on a single microtubule and intensity profile along the dashed line are shown, together with the corresponding fitting to the Gaussian function. Scale bar 2 μm; zoom-in 0.5 μm. The movies used for reconstruction are in Supplementary Videos 1 and 2. Fluorophore properties (histograms of localization uncertainty and photon count per molecule per frame and bleaching time-course) are shown. Fitting to log-normal distribution is shown as a thick line. Comparison of photon count per molecule per frame (c) and localization uncertainty (d) in living and in fixed samples. The data is from 6 and 7 fields of view, from 2 independently prepared samples.

**Figure 4:**
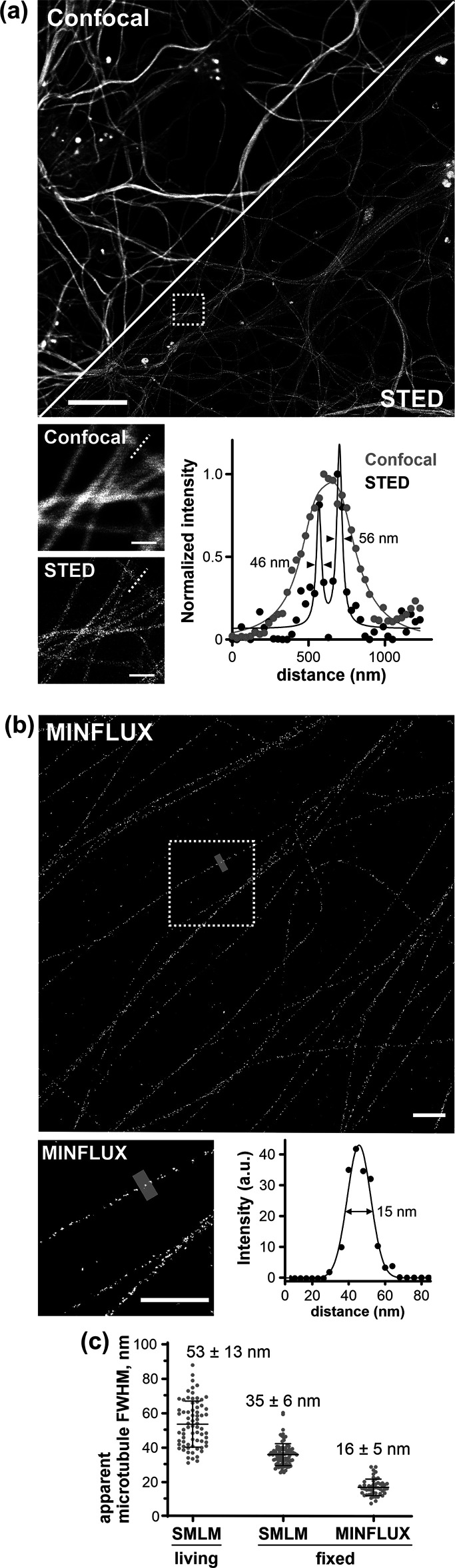
HMSiR-tubulin (**3**) performance in 2D-MINFLUX and 2D STED nanoscopy. (a) Confocal and 2D STED with 775 nm images of living mouse neurons stained with 300 nM HMSiR-tubulin (**3**). Scale bar 10 μm; inset 1 μm. Numbers represent fwhm. (b) MINFLUX image of microtubules in fixed U-2 OS cells stained with 20 nM HMSiR-tubulin (**3**) acquired in 3 h and rendered with 4 nm pixel size. Scale bar 500 nm. Line profile of the region (200 nm in width) shown in the inset. (c) Microtubule fwhm measured by SMLM and MINFLUX nanoscopy. MINFLUX data are from 5 fields of view, 2 independently prepared samples.

Next, we investigated the performance of HMSiR-tubulin (**3**) in SMLM imaging of microtubules in living and fixed cells. The image series of blinking fluorophores in living cells was acquired at 100 Hz frame rate in Hilo illumination mode (laser power of 0.4 kW cm^–2^). In the reconstructed images, the apparent diameter of the microtubule (fwhm) was 53 ± 13 nm (Figures 3a and 4c), which is smaller than 79 and 69 nm reported for HMSiR- and HMCR550-labeled HaloTag-tubulin, respectively.^12,24^ The small label size and its binding inside the microtubule cylinder are the likely reasons for a smaller apparent fwhm. Under these conditions, we were able to observe microtubule dynamics (i.e., growing and shrinking) over 4 min (Supplementary Video 3). The resolution in living cells is limited by a low photon number detected per molecule per frame. Increasing the laser power led to increased photobleaching, while longer exposure times resulted in too much motion blur. In fixed cells, imaging with 20 Hz increased the number of photons per molecule, and a microtubule fwhm of 35 ± 6 nm could be achieved (Figures 3b–d and 4c).

Both in living and in fixed cells, we saw little fluorescence recovery after photobleaching when prolonged illumination was alternated with incubation in the dark (Figure S8). This has been observed before with a nonblinking probe, SiR-tubulin,^25^ and can be explained by a high (millimolar) local concentration of taxane binding sites on the microtubule. Consistently with slow exchange, we were able to image fixed microtubules several hours after washing away the unbound probe.

A probe with a more prevalent dark state can be advantageous for imaging densely labeled structures like microtubule filaments. Thus, we examined probe **2** in the SMLM experiment. In fixed cells, it yielded images of similar quality as **3** (Figure S9). However, the reduced brightness of the probe made the choice of a region of interest in living cells extremely difficult. In summary, probe **3**, HMSiR-tubulin, showed the best staining and biocompatibility and is the probe of choice for SMLM of microtubules.

In addition, we found that HMSiR-tubulin can be used for 2D and 3D stimulated emission depletion (STED) nanoscopy of densely packed tubulin bundles in living neurons, although the brightness is low and high excitation laser power is required (Figure 4a and Supplementary Video 4). Similar to the previously reported DNA probes,^18^ we did not observe blinking. The fwhm of a microtubule determined by 2D STED was similar to that by SMLM (∼50 nm) (Figure 4a). In the *z*-axis, ∼2.5-fold improvement over standard confocal resolution was achieved (Figure S10). SiR-tubulin is preferable for STED imaging due to its substantially higher brightness. However, HMSiR-tubulin offers the possibility to image the same sample with STED and SMLM, which allows for confirmation of results by a different technique or standardizing SMLM reconstruction algorithms.

Next, we wanted to see if we could use probes **2** and **3** for MINFLUX imaging. MINFLUX nanoscopy localizes molecules with single digit nanometer precision^26,27^ by exploiting patterned excitation laser beam typically shaped as a donut. The spontaneous blinking ensured sparse distribution of fluorophores and the high contrast allowed us to record MINFLUX images of densely labeled microtubules with ∼2.3 nm localization precision (Figure 4b and S11). The very small size of the probes (∼2 nm) allows measurements without a strong linkage error. MINFLUX imaging enabled us to validate experimentally that HMSiR-tubulin (**3**) binds to microtubule filaments on the inner lumen, because the measured fwhm of a single filament of 16 ± 5 nm (Figure 4c) agrees very well with the inner diameter of the microtubule determined by cryo-electron microscopy (18 nm).^28^ A discontinuous appearance of microtubules in MINFLUX and SMLM images might result from the difficulty of localizing the individual fluorophores on the densely labeled structures or might reflect an incomplete preservation of binding sites or other interactors preventing probe binding.

In conclusion, we employed ligand and linker optimization to obtain HMSiR-tubulin, the first spontaneously blinking cell permeable probe for microtubules in living and fixed cells. It is compatible with different nanoscopy methods, SMLM, STED (with 775 nm STED laser), and MINFLUX, thereby allowing interchangeable use of these techniques. For the first time, we resolved the inner microtubule diameter by optical MINFLUX microscopy. We believe that this versatility will facilitate investigation of microtubule-related processes as well as developing microscopy imaging and data processing techniques.

## Methods

### Staining Living Cells

Living U-2 OS cells were incubated with 100 nM probe in DMEM medium with 10% FBS for 1 h at 37 °C and imaged without washing at RT.

### Fixing and Staining Cells

U-2 OS cells were fixed at RT as described.^5,20^ The cells were washed four times with 200 μL of PEMP (100 mM PIPES (pH 6.8), 1 mM EGTA, 2 mM MgCl_2_, and 4% PEG 8000), permeabilized for 90 s with 0.5% Triton X-100 in PEM (PEMP without PEG 8000), and washed again 4× with 200 μL of PEMP. Then, the cells were incubated with 200 μL of 0.2% glutaraldehyde in PEM for 15 min, followed by 200 μL of 2 mg mL^–1^ NaBH_4_ in PEM (dissolved immediately before use) for another 15 min. The samples were washed 4× with 200 μL of PEM and stained. For MINFLUX imaging, the samples were additionally incubated with gold nanoparticles (200 nm diameter, A11-200-CIT-DIH-1-10, Nanopartz) for 10 min and washed 4× with 200 μL of PEM.

The microtubules were stained with 10 nM (SMLM) or 20 nM (MINFLUX) probe in glycerol buffer (GB, 10 mM Na-PO_4_, pH 6.8, 1 mM EGTA, 6 mM MgCl_2_, 3.4 M glycerol) for 2–3 h at 4 °C and imaged without washing at RT.

### SMLM Imaging

The SMLM data was acquired on a Visitron Spinning disk/TIRF/SMLM system (Visitron Systems GmbH) equipped with Nikon CFI Apochromat TIRF 100×C Oil NA 1.49 objective and Prime 95B sCMOS camera (Teledyne Photometrics, pixel size 108 nm) in Hilo illumination mode with TIRF angle of 62°. A laser (0.4 kW cm^–2^, 640 nm) was used for excitation. Living cells were imaged at 100 Hz and 2500 (25 s) frames were used to reconstruct the image. Fixed cells were imaged at 20 Hz, and the images were reconstructed from 4500 frames (225 s).

The data was processed with SVI Huygens Localizer (version 20.10) by fitting each fluorescence event to the 2D Gaussian distribution. The localizations with uncertainty <50 nm and photon count <5000 for living or <10000 for fixed cells were made into histograms of 256 bins. After correcting for drift, the high resolution images were rendered with a pixel size of 5 nm and fixed fwhm of 15 nm. Mean localization uncertainty was calculated by fitting the uncertainty histogram to log-normal distribution in Origin 8.6.0. Mean photon number per molecule per frame was calculated as the mean of all localizations.

### MINFLUX Imaging

MINFLUX imaging was performed on an Abberior MINFLUX microscope (Abberior) equipped with a 1.4 NA 100× Oil objective lens as previously described.^29^ Images were acquired in 2D MINFLUX imaging mode using a 642 nm excitation laser (60.7 μW cm^–2^). Laser powers were measured at the position of the objective back focal plane using a Thorlabs PM100D power meter equipped with a S120C sensor head. All MINFLUX images display raw data and were rendered with Imspector (version 16.3.11657, Abberior) with 4 nm pixel size in histogram-mode, without any filtering or data processing.

## Supporting Information Available

Supplementary Figures S1-9, materials and methods, synthetic procedures, and characterization of the compounds (PDF)
Blinking of HMSiR-tubulin in living U-2 OS cells (AVI)
Blinking of HMSiR-tubulin in fixed U-2 OS cells (AVI)
Tubulin dynamics in living U-2 OS cells over 4 min (AVI)
3D STED imaging of tubulin in primary mouse neurons (AVI)

## Supplementary Material

cb1c00538_si_001.pdf

cb1c00538_si_002.avi

cb1c00538_si_003.avi

cb1c00538_si_004.avi

cb1c00538_si_005.avi
